# 2322. Challenges Faced by Perinatally-Infected Young People Living with HIV in Kenya During the COVID-19 Pandemic

**DOI:** 10.1093/ofid/ofad500.1944

**Published:** 2023-11-27

**Authors:** Manjot Singh, Winstone Nyandiko, Allison DeLong, Celestine Ashimosi, Dennis Munyoro, Janet Lidweye, Jack Nyagaya, Whitney Biegon, Josephine Aluoch, Ashley Chory, Edwin Sang, Eslyne Jepkemboi, Millicent Orido, Vladimir Novitsky, Rachel Vreeman, Rami Kantor

**Affiliations:** Alpert Medical School of Brown University, Providence, Rhode Island; Academic Model Providing Access to Healthcare (AMPATH), Eldoret, Kenya andDepartment of Child Health and Paediatrics, School of Medicine, College of Health Sciences, Moi University, Eldoret, Kenya, Eldoret, Rift Valley, Kenya; Brown University, Providence, Rhode Island; Academic Model Providing Access to Healthcare (AMPATH), Eldoret, Kenya., Eldoret, Rift Valley, Kenya; Academic Model Providing Access to Healthcare (AMPATH), Eldoret, Kenya., Eldoret, Rift Valley, Kenya; Academic Model Providing Access to Healthcare (AMPATH), Eldoret, Kenya., Eldoret, Rift Valley, Kenya; Academic Model Providing Access to Healthcare (AMPATH), Eldoret, Kenya., Eldoret, Rift Valley, Kenya; Academic Model Providing Access to Healthcare (AMPATH), Nairobi, Nairobi Area, Kenya; Academic Model Providing Access to Healthcare (AMPATH), Eldoret, Kenya., Eldoret, Rift Valley, Kenya; Arnhold Institute for Global Health, Icahn School of Medicine at Mount Sinai, New York, New York; Academic Model Providing Access to Healthcare (AMPATH), Eldoret, Kenya., Eldoret, Rift Valley, Kenya; Academic Model Providing Access to Healthcare (AMPATH), Eldoret, Kenya., Eldoret, Rift Valley, Kenya; Academic Model Providing Access to Healthcare (AMPATH), Eldoret, Kenya., Eldoret, Rift Valley, Kenya; Alpert Medical School of Brown University, Providence, Rhode Island; Icahn School of Medicine at Mount Sinai, New York, New York; Alpert Medical School of Brown University, Providence, Rhode Island

## Abstract

**Background:**

Understanding challenges experienced by young people living with HIV (YPLWH) during the COVID-19 pandemic could guide care during public health crises, particularly in resource-limited settings.

**Methods:**

Surveys utilizing validated and self-reported measures were administered to perinatally-infected YPLWH receiving care at four clinics at the Academic Model Providing Access to Healthcare (AMPATH) in western Kenya between February 2021 and July 2022. Reported psychological, physical, and socioeconomic challenges were summarized overall and across viral load (VL) thresholds (virologic failure VL >40 copies/mL; treatment failure VL >1000 copies/mL). COVID-19-related events, restrictions, and interventions in Kenya from the beginning of the pandemic through the entirety of the study period were compiled into a pandemic timeline and temporal patterns of the evaluated challenges within this timeline were assessed using generalized additive models.

**Results:**

Among 442 participants (median age 17 years, IQR 14-19; 49% female), 89% reported any challenge (mean 2.92 of the 23 challenges estimated; SD=2.14); 48% psychological, 66% physical, and 62% socioeconomic. Major psychological challenges included moderate to severe depression (36%) and anxiety (29%) symptoms. Major physical challenges included recent illness (31%), and recent symptoms of cough (34%), congestion (25%), and fatigue (23%). Major socioeconomic challenges included food insecurity (31%), household income loss (27%), and departure from usual residence (20%). Participants with virologic or treatment failure experienced more challenges compared to those suppressed. Trends of challenges over time were stable for all but physical challenges, which decreased in parallel with the reduced incidence of respiratory infections as masking and social distancing measures were implemented.

**Conclusion:**

The vast majority of Kenyan YPLWH experienced significant wellness challenges during the COVID-19 pandemic, conceivably due to pandemic-imposed travel restrictions and mitigation measures, and possibly impacting HIV care. This knowledge could guide intervention opportunities in this vulnerable population for current and future pandemics.

**Disclosures:**

**All Authors**: No reported disclosures

